# Case report: ^18^F-FDG PET/CT skeletal superscan-like in an adult patient with acute lymphoblastic leukemia

**DOI:** 10.3389/fonc.2024.1401453

**Published:** 2024-07-15

**Authors:** Yu Hu, Wenli Dai, Peng Wang, Yawen Feng, Hui Feng, Jun Li

**Affiliations:** Department of Nuclear Medicine, The First College of Clinical Medical Science, China Three Gorges University, Yichang, China

**Keywords:** acute lymphoblastic leukemia, ^18^F-FDG PET/CT, bone marrow, diffuse uptake, superscan

## Abstract

We herein describe a rare case of adult acute lymphoblastic leukemia with an ^18^florine-fluorodeoxyglucose (^18^F-FDG) positron emission tomography/computed tomography (PET/CT) skeletal superscan-like appearance. The degree of bone marrow uptake was so intense that it far exceeded the level of physiological cerebral uptake and radiourinary activity. The distribution was remarkably similar to a superscan seen on skeletal scintigraphy. Skeletal superscans of ^18^F-FDG PET/CT have been reported in hematological diseases, solid tumors with extensive bone metastasis, and metabolic diseases. Thus, we reviewed the PET/CT images of cases reported, indicating that more homogeneous distribution, without primary tumor and specific mandibular and skull activity, may be suggestive of hematological diseases.

## Introduction

Acute lymphoblastic leukemia (ALL) is one of the predominant subtypes of leukemia characterized (1). It is the most common malignancy in childhood with a typical age of presentation between 1 and 4 years and is rarely seen in adults (2). Clinical presentation can be nonspecific and span a spectrum of symptoms and signs. The majority of patients present with fever, pallor, and bruising as early signs of marrow suppression or hematologic abnormalities (3). It is difficult to accurately diagnose ALL using conventional imaging techniques (4). The National Comprehensive Cancer Network (NCCN) Clinical Practice Guidelines in Oncology have demonstrated the application value of positron emission tomography/computed tomography (PET/CT) in ALL (5). Some ALL can present with intense bone marrow uptake on an ^18^florine-fluorodeoxyglucose (^18^F-FDG) PET/CT skeletal superscan-like image, although superscans have been described in both benign and malignant diseases (6). In this case report, we present the unusual ^18^F-FDG PET/CT scan performance of a patient with ALL and review the relevant literature on this subject.

## Case presentation

**Figure 1:**
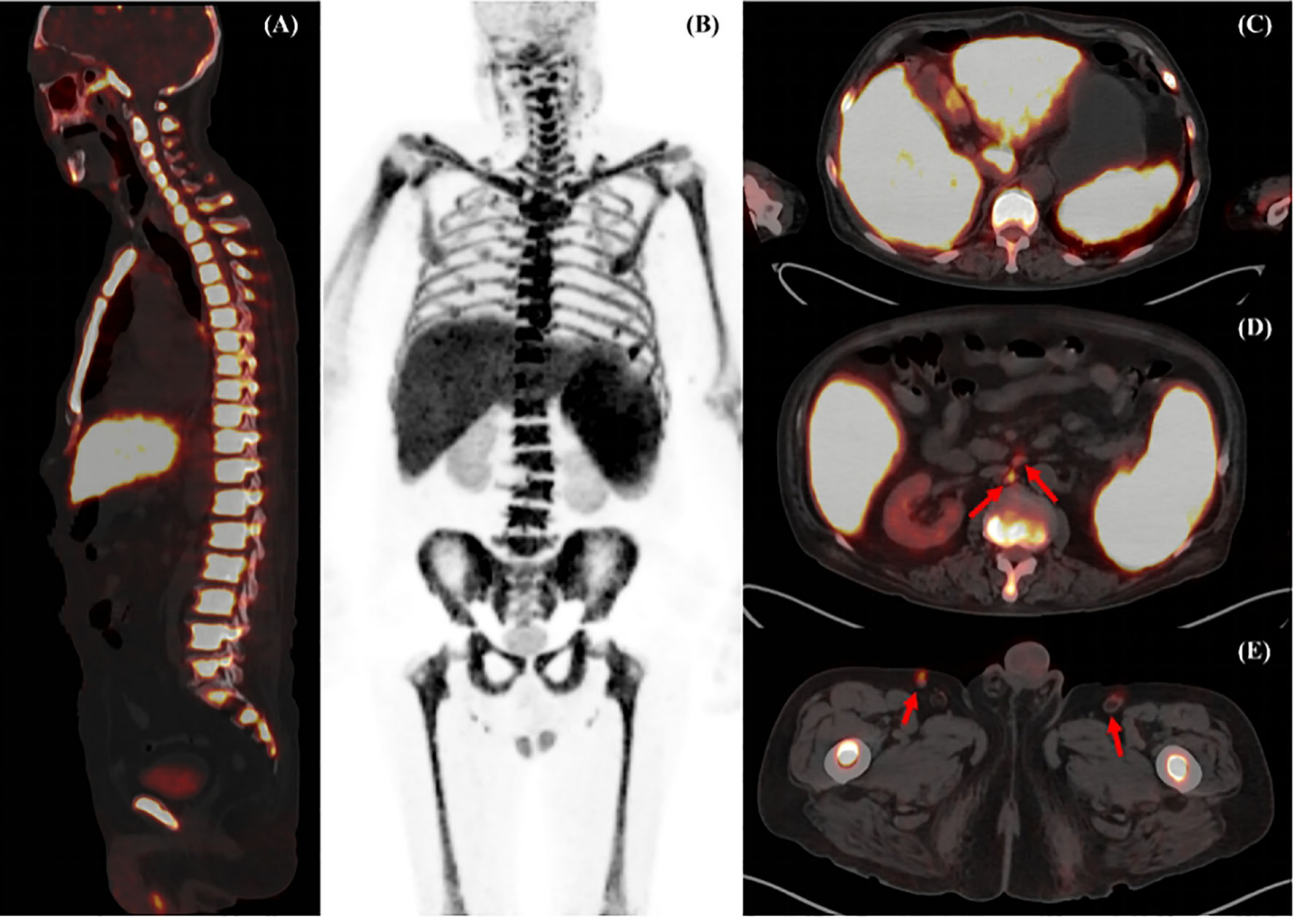
Maximum intensity projection (MIP) images of ^18^F-FDG PET/CT **(A)** showed markedly increased diffuse abnormal ^18^F-FDG uptake in the bone marrow of the axial and appendicular skeleton. Fusion PET/CT images **(B)** demonstrated the middle axis and sternum with high metabolic activity. Transverse fusion PET/CT showed increased uptake throughout the enlarged spleen and liver **(C)**, and revealed increased FDG uptake in slightly enlarged lymph nodes in the retroperitoneal and bilateral inguinal areas (**D, E**, arrow).

A 74-year-old male patient with a two-week history of fever, generalized weakness, and shortness of breath. The patient was a nonsmoker with no history of high blood pressure, diabetes, heart disease, familial inheritance, or malignancy. Laboratory examinations revealed decreased hemoglobin with 36g/L (normal range, 120-160 g/L), leukopenia (3.32 x 10^9^/L; normal range, 4-10 x 10^9^/L), thrombopenia (22 x 10^9^/L; normal range, 100-300 x 10^9^/L), total protein (48.61g/L; normal range, 63-85 g/L), albuminopenia (25.32g/L, normal range, 40-55 g/L), and A/G (1.09; normal range, 1.5-2.5). Furthermore, serum ferritin (2585ng/ml; normal range, 30-400 ng/ml), lactate dehydrogenase (570U/L: normal range, 0-248 U/L), urea (10.8 mmol/L; normal range, 2.86-7.14 mmol/L), crea (119 umol/L; normal range, 45-84 umol/L), and c-reactive protein (176.99mg/L; normal range, 0-10 mg/L) increased. The patient’s performance status (PS) score was 3. CT showed lung infection, irregular liver margins, and a markedly enlarged spleen. The patient received blood transfusions without cytokine release therapy or drug interference that could disturb bone marrow glucose metabolisms. The patient underwent an ^18^F-FDG PET/CT scan (**Figure 1**) and bone marrow biopsy for further diagnosis. Interestingly, the ^18^F-FDG PET/CT scan showed markedly increased diffuse uptake in axial and appendicular bone marrow with a maximum standardized uptake value (SUVmax) of 14.5 and also in the liver and spleen with a SUVmax 9.4 and 12.3, respectively. Furthermore, the brain (SUVmax 1.3), muscles (SUVmax 0.7), kidney (SUVmax 3.3), and bladder (SUVmax 3.2) were barely discernible as background activity. Axial sections of fused PET/CT images showed increased FDG uptake in slightly enlarged lymph nodes in the retroperitoneal and bilateral inguinal areas. The findings from the ^18^F-FDG PET/CT scan showed increased skeletal radiotracer uptake relative to soft tissues and absent or faint genitourinary tract activity suggestive of a superscan-like pattern. The results of bone marrow cytology 1 day later suggested ALL, which was consistent with the ^18^F-FDG PET/CT findings, although these were not specific and challenging in the initial diagnosis. Histopathology of the liver and spleen was not performed due to the patient’s poor basal status. After 7 days of hospitalization, the patient refused further tests and treatment and requested to be discharged. Unfortunately, his condition rapidly worsened and he expired within 1 day.

## Discussion

Acute leukemia (AL) has become a global health concern due to its increasing incidence over the past decade. Acute lymphoblastic leukemia is one of the most studied leukemias. It is characterized by a proliferation of lymphoid precursor cells in the bone marrow and other extramedullary sites. Diagnosing leukemia and assessing its potential spread to sites outside the bone marrow at early stages is essential for adequate management and mitigation of the complications. In recent years, more research reports have shown that ^18^F-FDG PET/CT also plays an important role in the diagnosis and treatment of ALL (7, 8).

In our patient, on an ^18^F-FDG PET scan, intense and homogeneous radiotracer uptake was observed in the axial and appendicular skeleton, and background activity in the genitourinary system, brain, and soft tissues was barely visible. The distribution of ^18^F-FDG showed a “superscan-like” appearance that was remarkably similar to that on the skeletal scintigraphy, showing an interesting phenomenon. The brain uptake is reportedly decreased in patients with extensive lymphoma lesions with the SUVmax of the brains of patients and controls was 13.1 ± 2.3 vs 14.9 ± 2.4 (9). In this case, the patient’s brain had very low activity with a SUVmax of 1.3, which is quite rare in the daily clinical practice.

The term “super bone scan” or “superscan” was first proposed by Osmond et al. in 1975, which was originally described as the appearance of the skeleton standing out “in bold relief” with faint radiotracer in the soft tissues and kidneys in a pattern on ^99^mTc-methylene diphosphonate skeletal scintigraphy (10). Later, Su et al. generalized the application of this term to similar features found in PET/CT (11). This feature “looks too good” due to the diffuse and symmetric skeletal uptake, similar to the appearance of a bone scan. To date, diffuse tracer uptake in the skeleton/bone marrow as a skeletal superscan pattern on ^18^F-FDG PET/CT has been previously reported in hematological diseases (including two cases of multiple myeloma (12, 13), two cases of lymphoma and two cases of acute lymphoblastic leukemia (6, 14–16), and one case of hemophagocytic lymphohistiocytosis) (17), nine cases of solid tumors with extensive bone metastasis (11, 18–25), and metabolic diseases with three cases of hyperparathyroidism and one case of renal osteodystrophy (26–29). By reviewing all the medical images of the reports, the hematological diseases revealed greater homogeneous radiotracer uptake in the skeleton, as in our case, and the metabolic diseases all had a specific skull and mandible intense uptake pattern. Thus, highly homogeneous radiotracer uptake in the skeleton without skull and mandible intense uptake and a primary tumor indicate hematological diseases, although it is difficult to determine the subtype.

In leukemia, ^18^F-FDG PET/CT is of use in diagnosing, staging, restaging, assessing extramedullary involvement, follow-up, and detecting Richter transformation (30). It is used especially in those people with nonspecific symptoms, such as fever and anemia of unknown origin, for detecting and monitoring extramedullary and unusual relapse sites and evaluating treatment response (30–33). It also plays an important role in the early detection of Richter’s syndrome (RS) and guiding biopsy in chronic lymphoblastic leukemia (CLL). Furthermore, it can also be used to diagnose graft versus host disease (GVHD) after allogeneic hematopoietic stem cell transplantation (allo-HSCT) (8). We found two case reports that reported patients with fever of unknown origin, and while examination of their peripheral blood showed no remarkable abnormalities, PET/CT revealed diffuse high ^18^F-FDG uptake in the bone marrow, suggesting the diagnosis of ALL (31, 33). In our case, the patient had a fever of unknown origin for two weeks, and the PET/CT scan indicated bone marrow abnormality without solid tumor uptake, which was suggestive of possible hematological disease.

In ALL, increased uptake of ^18^F-FDG in bone marrow may be a characteristic finding, including diffuse and focal patterns. However, increased bone marrow ^18^F-FDG uptake can also be observed in benign etiologies and other types of malignant infiltration, including following administration of growth factors, such as colony-stimulating growth factor or erythropoietin (32). Thus, it is difficult to differentiate. Some articles have reported that bone marrow malignant infiltration derived from lymphoma and leukemia generally has a markedly higher ^18^F-FDG uptake in the bone marrow compared with benign etiologies (34, 35).

Some studies focused on the prognostic value of PET/CT findings. Zhao et al. analyzed a total of 72 patients with acute leukemia and found that the presence of extranodal, extramedullary, and extrasplenic sites was identified as an independent prognostic indicator when treated with allogeneic hematopoietic stem cell transplantation treatment (36). The patient in our case died very soon. In the reported case of two ALL patients with superscan, one was reported dead two months after the PET/CT scan, suggestive of a considerably poor prognosis (16), while the outcome for the other was not reported. However, the sample was too small. The prognostic value of PET/CT remains to be explored.

In summary, the initial clinical presentation of ALL is variable and ^18^F FDG PET/CT is helpful in diagnoses, especially in those with a fever of unknown origin. The skeletal superscan-like appearance of ^18^F-FDG PET/CT is a rare phenomenon in clinical practice. It has been reported in hematological diseases, solid tumors, and metabolic diseases and the specific imaging patterns may help us to find the cause of illness.

## Data availability statement

The original contributions presented in the study are included in the article/supplementary material. Further inquiries can be directed to the corresponding author.

## Ethics statement

Ethical review and approval was not required for the study on human participants in accordance with the local legislation and institutional requirements. The patients/participants provided their written informed consent to participate in this study. Written informed consent was obtained from the individual(s) for the publication of any potentially identifiable images or data included in this article.

## Author contributions

YH: Data curation, Writing – original draft, Writing – review & editing. WD: Resources, Writing – review & editing. PW: Resources, Writing – review & editing. YF: Data curation, Writing – review & editing. HF: Supervision, Writing – review & editing. JL: Supervision, Writing – review & editing.
